# Hypothermia in neonates born by caesarean section at a tertiary hospital in South Africa

**DOI:** 10.3389/fped.2022.957298

**Published:** 2022-12-06

**Authors:** Mariambibi Patel, Neo Ramagaga, Danielle Kruger, Grace Lehnerdt, Imraan Mansoor, Lesedi Mohlala, Dylan Rendel, Fathima Zaheed, Mimie Jordaan, Mantoa Mokhachane, Firdose Lambey Nakwa, Ramatsimele Mphahlele

**Affiliations:** ^1^ University of the Witwatersrand, Faculty of Health Sciences, Johannesburg, South Africa; ^2^ District Clinical Specialist Team, Johannesburg Health District, Johannesburg, South Africa; ^3^ Department of Paediatrics and Child Health, School of Clinical Medicine, Faculty of Health Sciences, University of Witwatersrand, Johannesburg, South Africa

**Keywords:** hypothermia, neonate, caesarean section, season, birth weight

## Abstract

**Introduction:**

neonatal hypothermia has previously been noted in a large proportion of neonates born through Caesarean section at Chris Hani Baragwanath Hospital (CHBAH), yet no study in South Africa specifically explores the extent and severity of the threat of hypothermia to this population of neonates.

**Objectives:**

to describe the proportion and severity of neonatal hypothermia in infants born *via* Caesarean section at CHBAH as well as to document and describe possible contributing factors to neonatal hypothermia in this population.

**Methods:**

A neonatal unit's database records were reviewed for demographic information of patients and their mothers, clinical characteristics, body temperature and outcomes. Comparisons between normothermic and hypothermic neonates were performed.

**Results:**

Forty-one percent of neonates born *via* Caesarean section had hypothermia at birth, of whom 71%, 27% and 2% had mild, moderate and severe hypothermia, respectively. Prevalence of admission hypothermia was 42%. On average, neonates were born at term and were of normal birth weight. No maternal factors were found to be statistically significant. Bag-mask ventilation (BMV) and cardiopulmonary resuscitation (CPR) [3.4% vs. 0.7%, *p*-0.033; OR 2.67 (95% CI: 1.06–6.77)] and an elevated lactate [13.25 vs. 3.2 mmol/l, *p*-0.032; OR 1.13 (95% CI: 1.01–1.26)] were associated with hypothermia. In the multivariable logistic regression analysis hypothermia in neonates was associated with an elevated lactate.

**Conclusions:**

Prevalence of hypothermia in neonates born by Caesarean section is high and further prospective studies are required to elucidate the factors contributing to this.

## Introduction

The third sustainable development goal proposed by the World Health Organization speaks directly to improving healthcare. Surrogate measures for the healthcare of a country are the maternal and childhood mortality rates. Neonatal mortality in South Africa can be attributed to five main causes: prematurity, intrapartum events, infections, congenital abnormalities and miscellaneous causes (1). Hypothermia in neonates is known to contribute to neonatal mortality due to neonatal infections, prematurity and asphyxia (2).

Neonatal hypothermia was noted in a large proportion of neonates born through Caesarean section in data collected during a baseline audit for a Quality Improvement by the EMS services, yet no study in South Africa specifically explores the extent and severity of the threat of hypothermia to this population of neonates. A retrospective review at Chris Hani Baragwanath Academic Hospital (CHBAH) showed that hypothermia in neonates had a significant association with neonates born at 1 000 g (OR = 1.79), those requiring resuscitation (OR = 2.32) and, in contrast to other studies, were more likely to be born vaginally (OR = 1.48). This was proposed to be due to poor temperature control in delivery rooms as opposed to operating theatres and due to the fact that neonates born of Caesarean section were more likely to be attended to by a doctor, resulting in better control of temperature. The study also reports a higher mortality rate in hypothermic infants within a week of delivery (3). The severity of hypothermia in this study was not specified. This study thus aims to describe the proportion and severity of neonatal hypothermia in infants born *via* Caesarean section at CHBAH. The study also aims to document and describe possible contributing factors to neonatal hypothermia in the study population.

## Methodology

A retrospective descriptive study was undertaken at Chris Hani Baragwanath Academic Hospital (CHBAH) Neonatology Unit for the period 1 January 2018 to 31 December 2018. The CHBAH neonatology unit comprises of an 18 bed neonatal intensive care unit (NICU), a 48 bed high care area, a 100 bed standard care nursery (SCN), a 19 bed Kangaroo Mother Care (KMC) ward. Approval for the retrospective review of the CHBAH Neonatology Unit's database was granted by the Human Research Ethics Committee [HREC] (Medical) in July 2020. Ethics amendment was applied for and granted in March 2021.

The study population included all neonates born *via* Caesarean section at CHBAH and excluded patients born outside of CHBAH – for example transferred into the hospital or born before arrival (BBA) and those patients without any body temperature data available.

The data collected was secondary in nature, as it was collected from the Research Electronic Data Capture (REDCap) database of the CHBAH Neonatology Unit. The variables collected included: season of birth, birth weight, gestational age, body temperature, 1-minute APGAR score, neonatal biochemical parameters, indication for Caesarean section, resuscitation outcomes, neonatal illnesses as well as a number of maternal factors (age, parity, gravidity, race, HIV status, antenatal care received, antibiotics received, antenatal steroids administered and complications). Hypothermia was defined as an axillary temperature below 36.5 °C as per the World Health Organization (WHO) (4). This was further stratified into mild (36 °C–36.4 °C), moderate (32 °C–35.9 °C) and severe hypothermia (<32 °C). An axillary temperature within 30 min of birth was regarded as the birth temperature. The 4 seasons were defined as spring from 1 September to 30 November, summer from 1 December to 28 February, autumn from 1 March to 31 May and winter from 1 June to 31 August. A normal birthweight (NBW) was defined as a weight between 2.5 and 4.0 kilograms (kg), macrosomia as > 4.0 kg, low birthweight (LBW) as < 2.5 kg, very low birthweight (VLBW) < 1.5 kg and extremely low birthweight (ELBW) as < 1 kg.

Anonymised data was analyzed with aid of the statistical package Stata (version 16). Chi-square tests were used to determine statistical significance of categorical variables. Continuous variables were analyzed as normally or non-normally distributed using histogram functions. Statistical significance of continuous variables was determined by *t*-tests and Mann-Whitney *U* tests (Wilcoxon Rank Sum test) for normally and non-normally distributed variables respectively. Logistic regression models were used to determine odds ratios. For the multivariable regression model variables with a *p*-values <0.1 were included and those variables with few observations were excluded. The meconium aspiration variable was omitted from the linear regression due to no observations in the non-hypothermia group.

## Results

**Figure 1:**
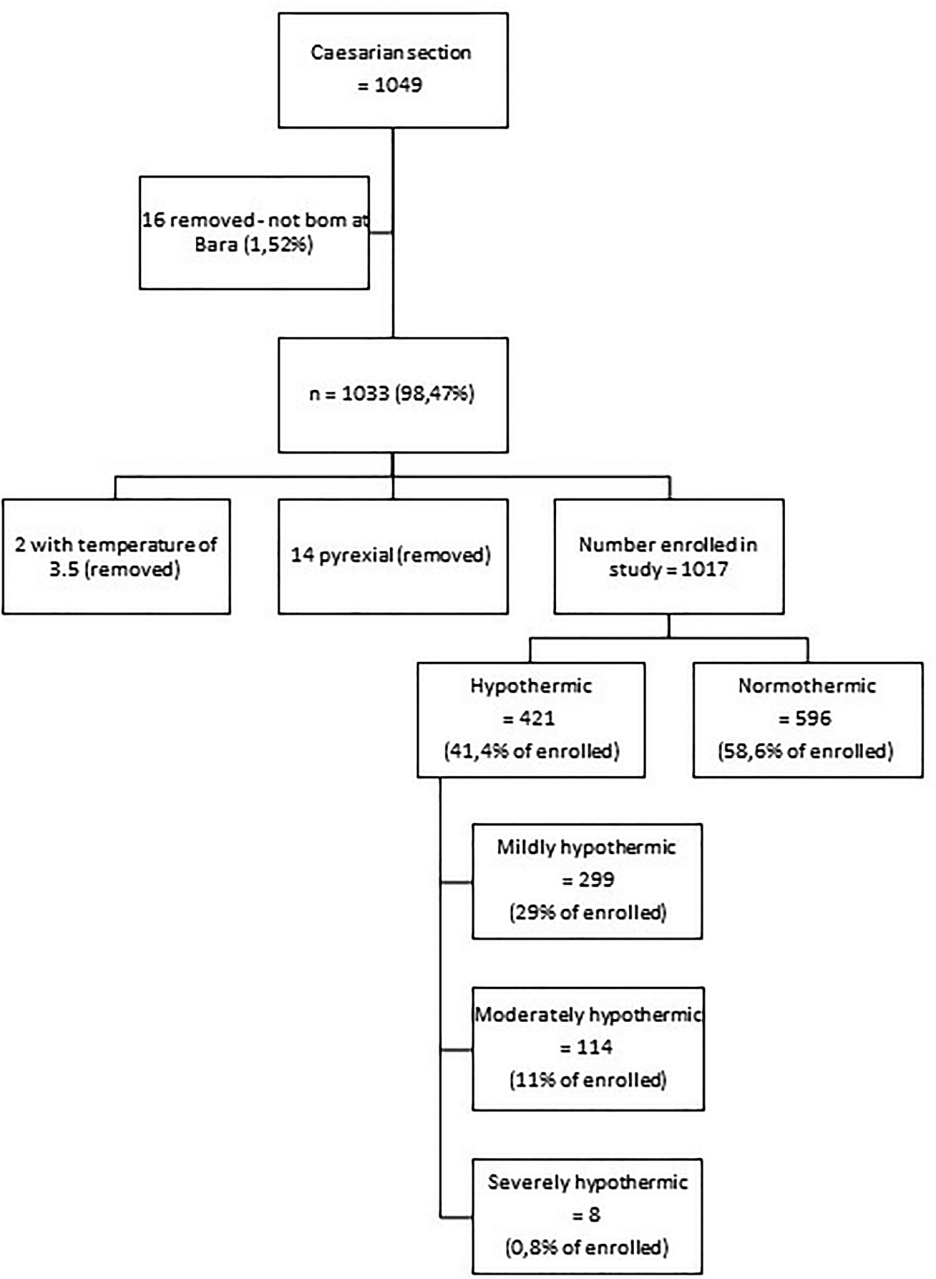
Consort diagram.

**Figure 2:**
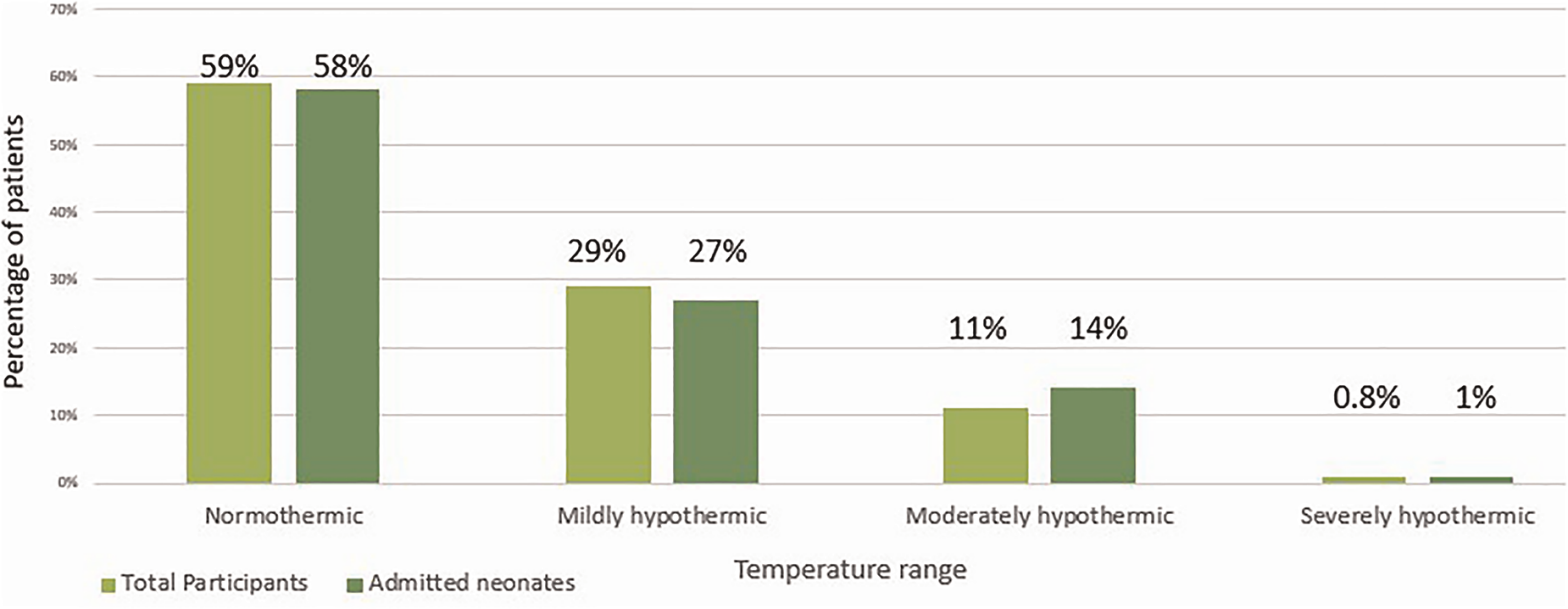
Comparison of severity of hypothermia amongst total and admitted neonates.

Of the 1,049 neonates born by Caesarean section at CHBAH during the study period, 1,017 (96.95%) were enrolled into the study (Figure 1). Forty-one percent (*n* = 421) had hypothermia at birth, of whom 71% (*n* = 299), 27% (*n* = 114) and 2% (*n* = 8) had mild, moderate and severe hypothermia, respectively (Figure 1). Additionally, of the 299 infants admitted to the neonatal wards at CHBAH, 42% (*n* = 125) were hypothermic (Figure 2). There were no significant differences when divided into mild, moderate and severe hypothermic groups. (Figure 2). The admitted infants were a subset of the study participants.

### Maternal characteristics

Most mothers in our study were African, with no Caucasians. They had a mean age of 28.4±6.6 years and were having their second child (mean 2±2). 25.3% were HIV positive and only 4.6% did not receive antenatal care. Receiving antibiotics, steroids or magnesium sulphate was not found to be associated with neonatal hypothermia (Table 1). The commonest indication for Caesarean section was foetal distress (64.7%). Neonates requiring Caesarean section for intrauterine growth restriction (IUGR) were less likely to be hypothermic [0% vs. 2.1%, OR 0.09 (95% CI: 0.00–0.56)] (Table 2).

**Table 1:** Maternal characteristics of study population.

Maternal factors	All *n*/*N* (%)	Hypothermic *n*/*N* (%)	Normothermic *n*/*N* (%)	*p*-value	Odds-ratio (95% CI)
Age*	28.4 ± 6.6	28.4 ± 6.6	28.5 ± 6.6	0.852	0.998 (0.98–1.02)
Parity#	1 (0–2)	1 (0–2)	1 (1–2)	0.238	0.94 (0.85–1.04)
Gravidity#	2 (1–3)	2 (1–3)	2 (1–3)	0.870	1.01 (0.91–1.11)
Unbooked	48/989 (4.6)	18/407 (4.4)	30/582 (5.2)	0.598	0.60 (0.33–1.08)
African Race	980/999 (98.1)	407/414 (98.3)	573/585 (98.0)	0.906	0.93 (0.62–1.39)
Maternal antibiotics	81/876 (9.2)	37/368 (10.1)	44/508 (8.7)	0.482	1.18 (0.74–1.87)
Maternal steroids	128/912 (14.0)	57/382 (14.9)	71/530 (13.4)	0.513	1.13 (0.78–1.65)
Maternal MgSO4	69/884 (7.8)	32/369 (8.7)	37/515 (7.2)	0.416	1.22 (0.75–2.00)

* Mean ± SD.

^#^ Median, IQR.

Missing values: Rh (*n* = 20), RVD (*n* = 25), RPR (*n* = 51), booked (*n* = 28), maternal antibiotics (*n* = 141), maternal steroids (*n* = 105), maternal MgSO4 (*n* = 133), parity (*n* = 5), gravidity (*n* = 7), age (*n* = 9).

**Table 2:** Indication for caesarean section.

Caesar Indication	All *N* = 881 *n* (%)	Hypothermic *N* = 362 *n* (%)	Normothermic *N* = 519 *n* (%)	*p*-value	ORs (95% CI)
Abnormal foetal presentation	25 (2.8)	962 (2.5)	16 (3.1)	0.600	0.80 (0.35–1.84)
Hypertensive disorders of pregnancy	84 (9.5)	40 (11.0)	44 (8.5)	0.201	1.34 (0.85–2.12)
Placental abnormalities	15 (1.7)	7 (1.9)	8 (1.5)	0.658	1.25 (0.45–3.50)
Abnormal labour	44 (5.0)	15 (4.1)	29 (5.6)	0.333	0.73 (0.39–1.38)
Foetal distress	570 (64.7)	242 (66.9)	328 (63.2)	0.264	1.17 (0.89–1.56)
CPD	35 (4.0)	17 (4.7)	18 (3.5)	0.359	1.37 (0.70–2.7)
Multiple pregnancies	41 (4.7)	11 (3.0)	30 (5.8)	0.057	0.51 (0.25–1.03)
Premature	7 (0.8)	3 (0.8)	4 (0.8)	0.924	1.08 (0.24–4.84)
Previous Caesar	125 (14.2)	47 (13.0)	78 (15.0)	0.392	0.84 (0.57–1.25)
IUGR	11 (1.2)	0 (0.0)	11 (2.1)	0.005	0.09 (0.0–0.56)
APH	32 (3.6)	14 (3.9)	18 (3.5)	0.755	1.12 (0.55–2.28)
Macrosomia	8 (0.9)	1 (0.3)	7 (1.3)	0.099	0.20 (0.02–1.65)

Missing:- *n* = 136.

Abnormal foetal presentation refers to breech, oblique lie, cord prolapse and face presentation.

Hypertensive disorders of pregnancy refers to PET, HELLP, Imminent eclampsia, PIH and chronic HT. Placental abnormalities refers to placental insufficiency, abruptio placentae, placenta previa, placenta accreta and chorioamnionitis. Abnormal labour refers to failed IOL, poor progress of labour, PROM, prolonged labour.

Bold values indicate statistical significance.

### Neonatal characteristics

**Figure 3:**
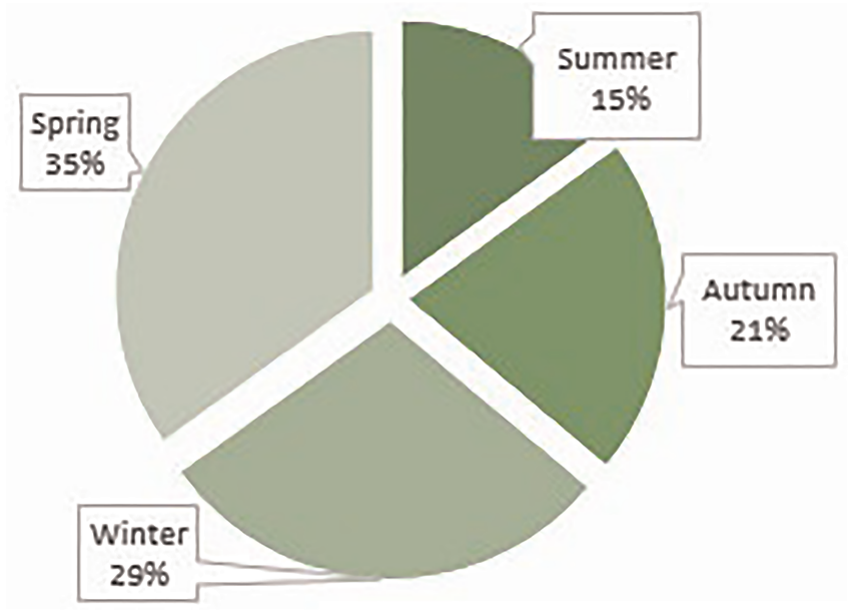
Seasonal variation amongst hypothermic neonates.

**Figure 4:**
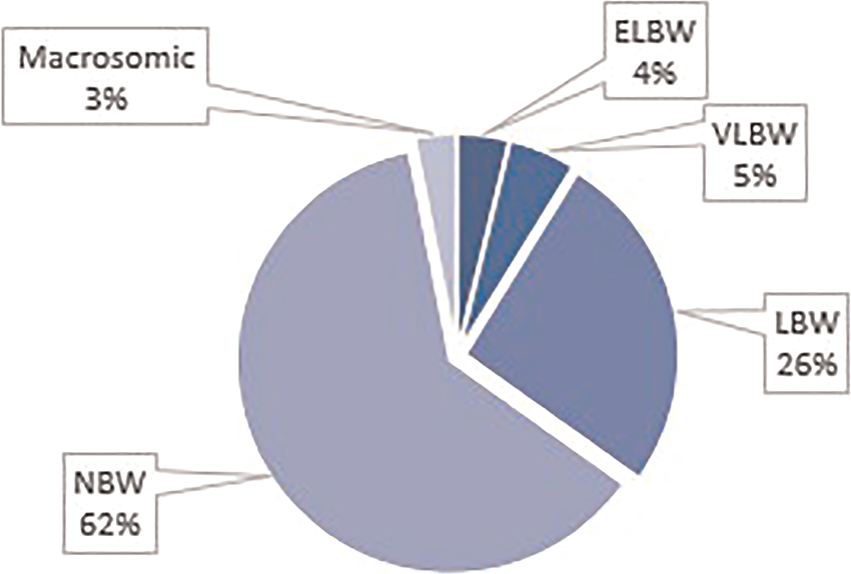
Weight distribution amongst hypothermic neonates.

Twenty-five percent of the study population (*n* = 251) were HIV exposed, 14% (*n* = 128) received antenatal steroids, 54% (*n* = 508) were male, 33% (*n* = 323) were premature and 21.9% (*n* = 169) were exposed to meconium. The mean gestational age and birth weight were 37.1 (±3.54) weeks, 2766.4 (±796.1) grams respectively. In 19.4% (*n* = 196) of neonates, the 1 min Apgar score was <7. Neonates who were exposed to meconium were less likely to be hypothermic [18.2% vs. 24.6%, OR 0.68 (95% CI: 0.48–0.99)] (Table 3). Hypothermic neonates were mostly born in spring followed by winter (Figure 3) and most were of normal birth weight (Figure 4). In addition, there were a larger number of deliveries in spring (5030) followed by winter (4799). Approximately 18.7% (*n* = 171) of all the neonates required resuscitation at birth (Table 4). Neonates who received both bag mask ventilation (BMV) and cardiopulmonary resuscitation (CPR) were more likely to be hypothermic than those who did not [3.4% vs. 0.7%, OR 2.67 (95% CI: 1.06–6.77)] (Table 4). However, other respiratory interventions such as nasal cannula, nasal continuous positive airway pressure (nCPAP), receiving surfactant or assisted ventilation were not significantly associated with the temperature of neonates. Being hypothermic increased the likelihood of having an elevated lactate [median lactate of 13.25 vs. 3.2 mmol/l, OR 1.13 (95% CI: 1.01–1.26)] (Table 5). Only a small proportion of neonates had an arterial blood gas (ABG) drawn as ABGs are only done in sick neonates.

**Table 3:** Characteristics of neonates enrolled in study.

Neonatal Characteristics	All *n*/*N* (%)	Hypothermic *n*/*N* (%)	Normothermic *n*/*N* (%)	*p*-value	Odds-ratio (95% CI)
Gestational age* (weeks)	37.1 ± 3.54	37.0 ± 3.62	37.2 ± 3.48	0.347	0.98 (0.95–1.02)
Birth weight (g)*	2766.4 ± 796.1	2749.5 ± 833.3	2778.3 ± 769.3	0.570	0.99 (0.99–1.00)
Season of birth (Winter)	267/1,017 (26.3)	122/421 (29.0)	145/596 (24.3)	0.207	1.08 (0.96–1.21)
Meconium exposure	169/772 (21.9)	59/325 (18.2)	110/447 (24.6)	0.033	0.68 (0.48–0.99)
Received resuscitation	171/913 (18.7)	98/534 (18.4)	73/379 (19.2)	0.729	1.06 (0.76–1.49)
APGAR 1 (<7)	196/1,009 (19.4)	87/417 (20.9)	109/592 (18.4)	0.589	1.60 (0.91–2.81)

* Mean ± SD.

Missing values: sex (*n* = 83), race (*n* = 18), Gestational age (*n* = 41), meconium exposure (*n* = 245), APGAR 1 (*n* = 8), APGAR 5 (*n* = 14), APGAR 10 (*n* = 530), birth weight (*n* = 6), resuscitation (*n* = 104). Bold values indicate statistical significance.

**Table 4:** Resuscitative measures undertaken.

Resuscitation details	All *N* = 913 *n* (%)	Hypothermic *N* = 379 *n* (%)	Normothermic *N* = 534 *n* (%)	*p*-value	Odds-ratio (95% CI)
BMV only	151 (16.5)	60 (15.8)	91 (5.5)	0.628	0.92 (0.64–1.31)
BMV and CPR	20 (2.2)	13 (3.4)	7 (0.7)	0.031	2.67 (1.06–6.77)
BMV, CPR and intubation	5 (0.5)	4 (1.1)	1 (0.1)	0.080	5.70 (0.63–51.07)
BMV, CPR, intubation and adrenaline	3 (0.3)	2 (0.5)	1 (0.1)	0.376	2.83 (0.26–31.10)

Missing (*n* = 104) BMV – bag mask ventilation, CPR – cardiopulmonary resuscitation Bold values indicate statistical significance.

**Table 5:** Neonatal biochemical parameters.

Neonatal Biochemical Factors	All Median (IQR)	Hypothermic Median (IQR)	Normothermic Median (IQR)	*p*-value	Odds- ratio (95% CI)
pH	7.26 (7.18–7.34)	7.24 (7.05–7.34)	7.26 (7.20–7.36)	0.54	0.26 (0.01–5.24)
pCO2	37.9 (29.2–48.0)	37.4 (29.2–53.2)	38.5 (29.6–42.8)	0.37	1.03 (1.00–1.06)
pO2*	107.2 (84.0–132.0)	107.4 (61.4–166.0)	111.0 (86.8–129.0)	0.98	1.00 (0.99–1.01)
Lactate	4.9 (2.5–15.0)	13.3 (5.4–19.7)	3.2 (1.9–5.1)	0.007	1.13 (1.01–1.26)
Base excess*	−8.2 ± 8.8	−9.5 ± 11.6	−7.3 ± 6.1	0.37	0.97 (0.91–1.04)

* Mean and Standard deviation values rather than median and IQR. Neonates that had arterial blood gases drawn: pH (*n* = 54), pCO2 (*n* = 56), pO2 (*n* = 54), base excess (*n* = 52), lactate (*n* = 33).

Bold values indicate statistical significance odds ratio results.

### Morbidity and mortality outcomes

Overall, 39.7% (*n* = 404) of the neonates were found to have a respiratory pathology, 2.5% (*n* = 25) were asphyxiated, 0.8% (*n* = 8) had a cardiac pathology, 1.0% (*n* = 10) had a congenital anomaly, 0.3% (*n* = 3) had subarachnoid haemorrhages and 2.0% (*n* = 20) were admitted for weight gain (Table 6). Neonates with hypothermia were less likely to have respiratory pathology (36.1% vs. 42.3%; *p*-0.047). Table 6. Thirty-one percent of (*n* = 299) neonates required admission, whereas 68% (*n* = 652) were discharged (Table 7). Only 0.2% (*n* = 2) neonates demised, 1 of which was hypothermic. It is believed that both deaths were stillbirths as their APGAR scores at 1 min were both 0.

**Table 6:** Neonatal diagnoses among study participants.

Neonatal diagnosis	All *N* = 1,017 *n* (%)	Hypothermic *N* = 421 *n* (%)	Normothermic *N* = 596 *n* (%)	*p*-value	Odds-ratio (95% CI)
Respiratory	404 (39.7)	152 (36.1)	252 (42.3)	0.047	0.77 (0.597–0.997)
Asphyxia	25 (2.5)	12 (2.9)	13 (2.2)	0.497	1.32 (0.59–2.91)
Cardiac	8 (0.8)	5 (1.2)	3 (0.5)	0.224	2.38 (0.56–1.00)
Congenital anomaly	10 (1.0)	3 (0.7)	7 (1.2)	0.462	0.60 (0.16–2.35)
Subarachnoid haemorrhage	3 (0.3)	1 (0.2)	2 (0.3)	0.776	0.71 (0.06–7.82)
Weight gain	20 (2.0)	8 (1.9)	12 (2.0)	0.898	0.94 (0.38–2.32)

Bold values indicate statistical significance odds ratio results.

**Table 7:** Neonatal outcomes among study participants.

Neonatal outcome	All *N* = 953 *n* (%)	Hypothermic *N* = 401 *n*/*N* (%)	Normothermic *N* = 552 *n* (%)	*p*-value	Odds-ratio (95% CI)
Neonatal outcome				0.953	1.02 (0.78–1.34)
Admit	299 (31.4)	125 (31.2)	174 (31.5)		
Discharged	652 (68.4)	275 (68.6)	377 (68.3)		
Death	2 (0.2)	1 (0.2)	1 (0.2)		

Missing (*n* = 64).

### Factors associated with hypothermia

Having had CPR, respiratory pathology, and an elevated lactate were associated with hypothermia. In the multivariate logistic regression model, neonates who were hypothermic were more likely to have an elevated lactate. (Table 8). Maternal factors were not found to significantly contribute to neonatal hypothermia.

**Table 8:** Factors associated with neonatal hypothermia.

Factors associated with hypothermia	Unadjusted Odds-ratio (95% CI)	*p*-value	Adjusted Odds-ratio (95% CI)	*p*-value
Meconium exposure	0.68 (0.48–0.99)	0.033	–	–
Respiratory pathology	0.77 (0.597–0.997)	0.048	4.35 (0.418–45.3)	0.219
BMV and CPR	2.67 (1.06–6.77)	0.038	0.409 (0.45–3.67)	0.425
Lactate	1.13 (1.01–1.26)	0.032	1.17 (1.02–1.34)	0.024

Variables with few observations were excluded from the multivariable regression model.

Variables with a *p*-values <0.1 were used to build the multivariate regression model.

The meconium aspiration variable was omitted from the linear regression due to no observations in the non-hypothermia group.

BMV- bag mask ventilation, CPR – cardiopulmonary resuscitation.

## Discussion

Most of the hypothermic patients in our cohort had mild hypothermia. Incidence of hypothermia in our study population and those neonates admitted were generally comparable. Neonates who were moderately and severely hypothermic had higher admission rates though this was not significant.

Only a small number of neonates required an arterial blood gas, with neonates who were hypothermic being more likely to have an elevated serum lactate. Elevated serum lactate results from anaerobic respiration, when oxygen demand is higher than the oxygen supply that the body is receiving. Therefore, low body temperature may reflect thermoregulatory failure due to insufficient energy secondary to a lack of oxygen or metabolite (5). Of the 13 neonates with elevated lactate and hypothermia, 11 had an APGAR score at 1 min less than 7 (84.6%). Hence, they received resuscitation and therefore had an element of encephalopathy; however, only 2 met the definition for intrapartum asphyxia; as the other neonates improved their APGAR scores at 5 min after resuscitation. Lactate takes longer than pH to normalize (6). Our study design did not allow for ascribing causation between hypothermia and higher lactate levels but the association is interesting to note.

Neonates who were hypothermic were more likely to have an elevated lactate. Hypothermia may be associated with hypoxia, poor perfusion and metabolic acidosis (2). This causes a build up of lactate in hypothermic neonates. In the current study hypothermic neonates were not hypoxic but had a metabolic acidosis which was not significant. Other causes of a high lactate would need to be investigated.

BMV together with CPR as a means of resuscitation at birth was also associated with hypothermia (*p* = 0.031). A possible explanation for this could be that during emergency resuscitations, neonates were exposed for adequate chest compressions to be delivered. This finding is similar to a study done in Bangladesh (OR = 2.43) (7) and a study done in Iran (OR = 1.91) (8).

Patients with respiratory pathology are closely monitored, as they often go on to require ventilation and admission to ICU. For this reason, it is believed that they were found to be less hypothermic in this study as they were likely kept warmer as a general measure. A study in Denmark found that neonates with hypothermia had an increase odds of having respiratory distress syndrome and bronchopulmonary dysplasia, however when these results were adjusted for confounders the findings were not significant (9). Complications of respiratory distress syndrome, need for respiratory support and an increase in admission to the NICU or standard care nursery are seen with a higher prevalence in neonates who are hypothermic (10, 11).

Studies have shown that neonates who are small for gestational age or low birthweight are more prone to hypothermia. Reasons cited for this are large head to body surface area and decreased insulation due to decreased body fat (10). Surprisingly neonates with IUGR were less likely to be hypothermic in this study although the group sizes for this comparison were very small. Merazzi et al. reported similar findings; where low birth weight neonates (< 2500 g) was not significantly associated with hypothermia and suggest that birth weight has a smaller effect on body temperature in healthy term and late preterm neonates (11). Heat loss in neonates occurs *via* 4 mechanisms which includes evaporation. Transepidermal water loss (TEWL) is inversely proportional to the gestational age with low TEWL in term neonates. Preterm neonates who are SGA have lower TEWL than preterm neonates who are appropriate for gestational age (12). Neonates with IUGR have a more developed dermis and epidermis and may be more equipped to maintain homeostasis as the skin barrier may be fully developed. We hypothesize that IUGR neonates have more mature skin and less subcutaneous fat and are thus less likely to lose heat *via* TEWL. The number of IUGR neonates are quite small in this study, thus it is difficult to make meaningful conclusions. Further studies are required to elucidate this hypothesis.

This study also evaluated the common factors known to be associated with neonatal hypothermia such as low birth weight, prematurity and season in which the neonate was born. In this study most neonates had a normal birth weight and were not premature. This could be due to the sample population selected as a high proportion of neonates who were born *via* Caesarean section were term neonates. Neonates in this study were mostly born in spring followed by winter. This could be explained by a seasonal variation in the number of deliveries with a higher proportion of neonates born in spring and winter, thus giving a relatively higher proportion of neonates being hypothermic. In addition, spring temperatures in Johannesburg can be as low as 8 to 12 °C (13).

The limitations of the study include the retrospective study design and missing variables. Thus, the study was not powered to fully elucidate the associated factors of hypothermia. The time at which temperatures was taken were not documented. Given that sicker neonates were more likely to have an ABG drawn, this lends itself to a selection bias in terms of the biochemical parameters documented.

## Conclusion

Hypothermia is a significant contributor to morbidity and mortality in many settings and mild hypothermia is often prevalent in the setting of Caesarean delivery. Receiving CPR and having an elevated lactate was associated with hypothermia in this cohort. During resuscitation more concerted efforts should be put in to place to prevent hypothermia. There is a need for future studies investigating hypothermia in neonates born *via* Caesarean section including a non-Caesarean section control group.

## Data Availability

The raw data supporting the conclusions of this article will be made available by the authors, without undue reservation.
